# Metabolomic Insights into Volatile Profiles and Flavor Enhancement of Spice-Smoked Chicken Wings

**DOI:** 10.3390/foods14132270

**Published:** 2025-06-26

**Authors:** Yajiao Zhao, Ye Guo, Danni Zhang, Quanlong Zhou, Xiaoxiao Feng, Yuan Liu

**Affiliations:** 1 Department of Food Science & Technology, School of Agriculture & Biology, Shanghai Jiao Tong University, Shanghai 200240, China; zhaoyj0820@sjtu.edu.cn (Y.Z.); g18042692029@163.com (Y.G.); dannizhang2019@sjtu.edu.cn (D.Z.); qlal200007@163.com (Q.Z.); 2 College of Food Science and Technology, Jiangxi Agricultural University, Nanchang 330045, China; 3 School of Food Science and Engineering, Ningxia University, Yinchuan 750021, China

**Keywords:** volatile organic compounds (VOCs), spice smoking, relative odor activity value (ROAV), metabolomic, household-friendly cooking

## Abstract

Traditional smoking techniques, while historically valued for preservation and flavor enhancement, face limitations in aromatic diversity and safety, prompting exploration of spice-derived alternatives to meet modern culinary demands. This study explores the volatile compound profiles and aroma modulation of chicken wings smoked with four spices—cardamom, rosemary, mint, and rose—using a novel, household-friendly smoking protocol. The method combines air fryer pre-cooking (180 °C, 16 min) with electric griddle-based smoke infusion, followed by HS-SPME/GC-TOF/MS, relative odor activity value (ROAV) calculations, and metabolomic analysis. A total of 314 volatile compounds were identified across five samples. Among them, 45 compounds demonstrated odor activity values (ROAV) ≥ 1, contributing to green, woody, floral, and sweet aroma attributes. Eucalyptol displayed the highest ROAV (2543), underscoring its dominant sensory impact. Metabolomic profiling revealed a general upregulation of differential volatiles post-smoking: terpenes were enriched in wings smoked with cardamom, rosemary, and mint, while aldehydes and alcohols predominated in rose-smoked samples. An integrated screening based on ROAV and metabolomic data identified 24 key volatiles, including eucalyptol, β-myrcene, methanethiol, and α-pinene, which collectively defined the aroma signatures of spice-smoked wings. Spice-specific aroma enrichment and sensory properties were evident: rosemary intensified woody–spicy notes, mint enhanced herbal freshness, and rose amplified floral attributes. The proposed method demonstrated advantages in safety, ease of use, and flavor customization, aligning with clean-label trends and supporting innovation in home-based culinary practices. Moreover, it facilitates the tailored modulation of smoked meat flavor profiles, thereby enhancing product differentiation and broadening consumer acceptance.

## 1. Introduction

Smoking is one of the oldest techniques for food preservation and flavor enhancement [1,2], traditionally involving the combustion of hardwoods to impart smoky, savory, and aromatic characteristics [3]. While originally intended to extend shelf life, smoking has evolved into a method of culinary expression focused on sensory appeal [4]. Today, traditional smoking materials such as oak and hickory dominate industrial and artisanal practices but offer limited aromatic diversity [5,6]. This has driven interest in alternative smoke sources—particularly spices—due to their rich volatile profiles. Spice-based smoking not only reduces the risk of harmful compounds associated with wood smoke but also enables the tailored development of floral, fruity, or herbal flavor notes, meeting modern consumer demands for innovation and variety [7].

However, conventional smoking techniques are time-consuming, equipment-intensive, and unsuitable for most home environments [8,9]. They often require precise control over temperature, humidity, and smoke density and pose health concerns due to the potential formation of harmful compounds like polycyclic aromatic hydrocarbons (PAHs) [10]. To overcome these limitations, this study proposes a simplified, home-adapted smoking protocol using common kitchen appliances. The procedure involves pre-cooking marinated chicken wings in an air fryer for rapid, oil-free heating and consistent texture, followed by spice-based smoking on a portable electric griddle enclosed in aluminum foil. This setup creates a sealed environment for efficient volatile transfer without specialized equipment. The approach is modular, allowing for a wide range of spice combinations across different culinary traditions. By enhancing accessibility and customization, the protocol aligns with current DIY cooking trends and offers a practical, safe, and versatile solution for flavor innovation in home kitchens.

Chicken wings, as a globally popular food item, are characterized by their high fat content, structural suitability for smoking, and broad consumer appeal. The fatty skin and porous muscle tissue of wings facilitate the retention of volatile compounds, while their relatively small size allows for rapid flavor penetration during both marination and smoking processes [11]. These features make chicken wings an ideal model for investigating the potential of flavor customization, particularly in the context of novel spice-based smoking applications [12]. In this study, four spices—cardamom, rosemary, mint, and rose—were selected based on their distinct aroma profiles, capacity for flavor enhancement, and consumer appeal. Cardamom (*Elettaria cardamomum*), rich in terpenes such as eucalyptol and limonene, imparts citrusy, sweet, and slightly minty notes and is widely used in Middle Eastern and South Asian cuisines [13]. Rosemary (*Rosmarinus officinalis*), characterized by compounds such as camphor, α-pinene, and borneol, contributes woody, pine-like aromas commonly favored in Mediterranean dishes [14]. Mint (*Mentha*), dominated by menthol and menthone, delivers a fresh and cooling sensation, integral to beverages and desserts [15]. Rose (*Rosa damascena*), abundant in terpenes, alcohols, and aldehydes, offers delicate floral and honey-like undertones, prevalent in confectionery and perfumery [16]. Despite their aromatic differences, their spices share low thresholds for key odorants, ensuring perceptible aroma contributions even at low concentrations. Their widespread global usage and flavor-enhancing properties underscore their potential to diversify smoked food offerings and appeal to consumers seeking novel sensory experiences.

This study aims to systematically evaluate the volatile compound profiles and aroma attributes of chicken wings smoked with cardamom, rosemary, mint, and rose, using a household-adapted smoking protocol. To this end, headspace solid-phase microextraction (HS-SPME) coupled with gas chromatography time-of-flight mass spectrometry (GC-TOF/MS) was employed for volatile identification. The relative odor activity value (ROAV) was calculated to estimate the sensory contribution of individual compounds, and metabolomic tools were used to analyze differential volatiles. Collectively, these approaches aim to validate the effectiveness of the air fryer–griddle smoking method for home use while offering practical insights for the optimization of spice-based flavor systems.

## 2. Materials and Methods

### 2.1. Samples and Chemicals

Dried products of four spices (cardamom, rosemary, mint, and rose) and chicken wings were purchased from a supermarket in Minhang District, Shanghai. The four spices were sealed and stored at room temperature, and the chicken wings were frozen at −20 °C. 2-Octanol (GC grade, ≥99.5%, used as an internal standard) was purchased from Aladdin Co., Ltd. (Shanghai, China). n-Alkane (C_7_–C_40_) was purchased from Sigma-Aldrich Co., Ltd. (Shanghai, China). Methanol (GC grade, ≥99.9%) was purchased from Beijing InnoChem Science & Technology Co., Ltd. (Beijing, China).

### 2.2. Processing of Four Spice-Smoked Chicken Wings

**Figure 1:**
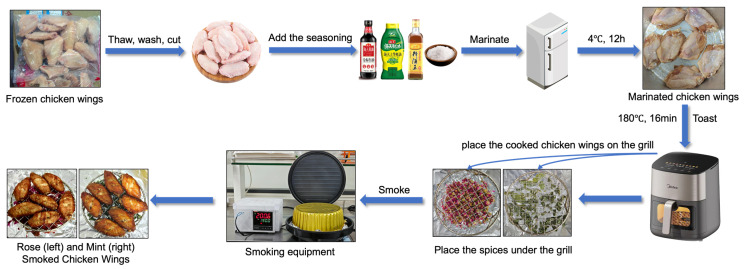
Schematic diagram of the smoking process for chicken wings.

The schematic diagram of the chicken wing smoking process is presented in Figure 1. After thawing, the chicken wings were immediately washed and cut. A mixture comprising light soy sauce, oyster sauce, and cooking wine (each at 2% of the chicken wings’ weight) and salt (0.05%) was then added. Following marination at 4 °C for 12 h, the chicken wings were cooked in an air fryer at 180 °C for 16 min. A smoking device was then assembled by placing various spices on a sheet of aluminum foil, positioning a grill above the spices, placing the cooked chicken wings on the grill, and covering the setup with an aluminum foil box to create a sealed smoking chamber. After smoking, the chicken wings were allowed to stand for 5 min. Subsequently, 5 g of the lower skin, in close proximity to the spices, was collected and transferred into a 20 mL headspace vial for analysis. The control group consisted of smoked chicken wings prepared without any added spices. Samples labeled CMW, RMW, MW, and RW corresponded to chicken wings smoked with cardamom, rosemary, mint, and rose, respectively.

### 2.3. HS-SPME/GC-TOF/MS Analysis

Analysis of volatile compounds was performed using HS-SPME combined with GC-TOF/MS, referring to the method of Qian et al. with slight modifications [17]. For HS-SPME analysis, a manual HS-SPME holder coupled with a 50/30 μm divinylbenzene/carboxen/polydimethylsiloxane (DVB/CAR/PDMS) fiber (Supelco, Bellefonte, PA, USA, the fiber length is 1 cm) was used. Add a 3.00 g sample to a 20 mL headspace vial with 10 μL internal standard and seal with a screw. The sample vial was equilibrated for 10 min in a 50 °C water bath, followed by fiber exposure to the headspace over the sample for 30 min.

After extraction by the HS-SPME-based procedure, the analytes were identified using an Agilent GC 7820A (Agilent Co., Santa Clara, CA, USA) equipped with a Pegasus BT time-of-flight mass spectrometer (LECO, St. Joseph, MI, USA). The separation occurred on an HP-INNOWAX capillary column (30 m × 0.25 mm, 0.25 μm). The carrier gas flow (helium, percentage purity > 99.999%) was 1.0 mL/min, constant flow. The injection port temperature was 250 °C, and a non-split injection was performed. The temperature program was as follows: the initial temperature was held at 40 °C for 3.0 min and then ramped to 250 °C at 5 °C/min and held for 10.0 min. The mass spectrometer was operated in an electron-impact mode of 70 eV, with a mass scan range of 35–550 amu (*m*/*z*). The temperatures of the interface and ion source were 290 and 230 °C, respectively.

### 2.4. Qualitative and Quantitative Analysis of Volatile Compounds

The qualitative method of volatile compounds is as follows: After excluding the impurity peaks, a series of C_7_–C_40_ n-alkanes were used as carbon standards to calculate the retention index (RI) of the target volatile compounds, and the mass spectra (MS) and retention index (RI) were compared with the data in the NIST 17 GC-MS database to screen compounds with a similarity greater than 700. The calculation of the retention index is shown in Formula (1):

(1) $$RI=\left(\frac{lgt_{i}-lgt_{n}}{lgt_{n+1}-lgt_{n}}+n\right)\times 100$$

where RI is the retention index of volatile compounds; n is the carbon number of n-alkane; *t*_n_ is the retention time of n-alkane with n carbon atoms; *t*_n+1_ is the retention time of n-alkane with n + 1 carbon atoms; and *t_i_* is the retention time of compound *i* to be tested.

The quantitative method of volatile compounds is as follows: Using the internal standard method, the internal standard was 2-octanol (500 μg/mL), and the mass concentration of each compound in the sample was calculated based on the mass concentration of the internal standard. The quantitative formula is shown in Formula (2):

(2) $$C_{x}=\frac{A_{x}\times C_{0}}{A_{0}}$$

where *C_x_* is the concentration of volatile compounds, μg/kg; *A*_0_ is the peak area of the internal standard compound 2-octanol; *A_x_* is the peak area of volatile compounds; and *C*_0_ is the concentration of the internal standard compound 2-octanol, μg/kg.

### 2.5. Calculation of Relative Odor Activity Value (ROAV)

ROAV is the ratio of the concentration of a volatile compound to its threshold value, and the calculation formula is as follows (3):

(3) $${\mathrm{ROAV}}_{i}=\frac{C_{i}}{OT_{i}}\times \frac{1}{1000}$$

where ROAV*_i_* is the odor activity value of compounds *i*; *C_i_* is the concentration of compound *i* in the spice (μg/kg); and OT*_i_* is the olfactory threshold of the compound in aqueous solution (mg/kg) [18].

### 2.6. Statistical Analysis

One-way analysis of variance (ANOVA) followed by Duncan’s multiple tests was performed to determine the differences between samples using SPSS (version 29.0, Chicago, IL, USA), where *p* < 0.05 was considered to be significant. Results were presented as the mean ± standard deviation (SD). Principal component analysis (PCA), Venn diagram, cluster heat map, and OPLS-DA analysis were performed using the Metware Cloud (https://cloud.metware.cn), accessed on 13 June 2025. Part of the data was visualized using GraphPad Prism (version 10, San Diego, CA, USA).

## 3. Results and Discussion

### 3.1. Volatile Profiles of Chicken Wings Before and After Smoking with 4 Spices

**Figure 2:**
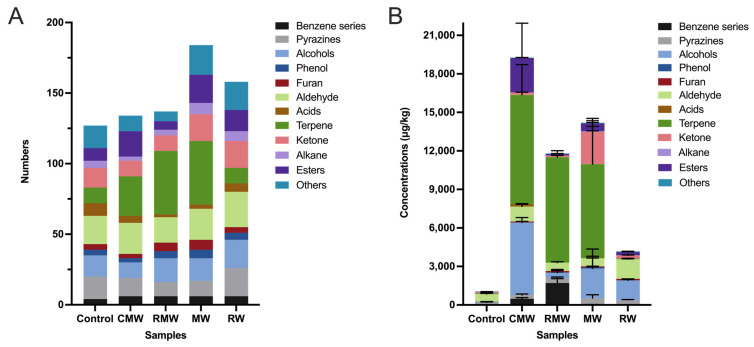
(**A**) Total amount differences in 12 groups of volatile compounds in chicken wings before and after smoking with four spices. (**B**) Total concentration differences in 12 groups of volatile compounds in chicken wings before and after smoking with four spices. Control represents smoked chicken wings without spices; CMW, RMW, MW, and RW represent smoked chicken wings with cardamom, rosemary, mint, and rose, respectively.

A total of 314 volatile compounds were tentatively identified across five chicken wing samples, including those before smoking (control) and after smoking with four different spices. These compounds were classified based on their chemical structures into the following categories: benzene derivatives (10), pyrazines (20), alcohols (31), phenols (9), furans (8), aldehydes (36), acids (10), terpenes (74), ketones (32), alkanes (14), esters (39), and others (31) (Table S1). The number of volatile compounds detected in the control, CMW, RMW, MW, and RW samples was 128, 133, 137, 185, and 165, respectively (Table S1). As shown in Figure 2A, the control sample contained 127 volatile compounds, whereas the spice-smoked samples ranged from 134 to 185, indicating an overall increase in volatile compounds following smoking. Among them, the MW sample exhibited the largest increase, with 58 additional volatiles detected. Notably, the number of acids decreased, while the number of terpenes increased in three spice-smoked samples. The most significant increases in terpene number were observed in the RMW and MW samples, with 34 compounds added in both. As illustrated in Figure 2B, the total concentration of volatile compounds in the control sample was 1046.24 ± 91.33 μg/kg, while in the four spice-smoked samples, concentrations ranged from 4172.88.16 ± 189.03 μg/kg to 19,266.46 ± 5818.15 μg/kg. The overall concentration of volatiles increased significantly upon spice smoking, with the most substantial increase observed in the CMW sample, reaching approximately 18 times that of the control. Terpenes and alcohols were dominant in all samples. In particular, terpene concentrations in the CMW and RMW samples increased by approximately 159-fold and 153-fold, respectively, while alcohol concentrations increased by 119-fold and 48-fold in CMW and MW, respectively.

Regarding individual compound concentrations, aldehydes and pyrazines were predominant in the control (non-spice smoked) sample, including isovaleraldehyde, hexanal, 2-methylbutanal, isobutyraldehyde, 2,5-dimethylpyrazine, and trimethylpyrazine (Table S1). The types of volatile compounds identified in this study were generally consistent with those reported in other smoked chicken samples, although concentrations varied [19]. In the cardamom–smoked chicken wings (CMW), the dominant volatiles were primarily terpenes, such as eucalyptol, limonene, and D-limonene. A similar profile was observed in the rosemary-smoked wings (RMW), where the major volatiles included eucalyptol, camphol, camphor, limonene, and α-pinene. In mint-smoked samples (MW), characteristic mint aroma compounds such as levomenthol were detected, along with isomenthone, ethanol, and piperitone, which were predominantly terpenes and ketones. In contrast, rose-smoked wings (RW) exhibited higher concentrations of aldehydes, including 2-methylbutanal, furfural, isovaleraldehyde, and isobutyraldehyde, as well as phenylethyl alcohol, a compound with a characteristic rose aroma.

Overall, both the types and concentration of volatile compounds increased significantly in chicken wings smoked with different spices. The dominant volatiles in each sample were largely consistent with the characteristic aroma profiles of the respective spices used (unpublished data) [14,16]. These findings preliminarily suggest that the family-friendly smoking method described in this study effectively imparts spice-derived aroma compounds to chicken wings.

### 3.2. Difference Analysis of Volatile Compounds in Four Spice-Smoked Chicken Wings

**Figure 3:**
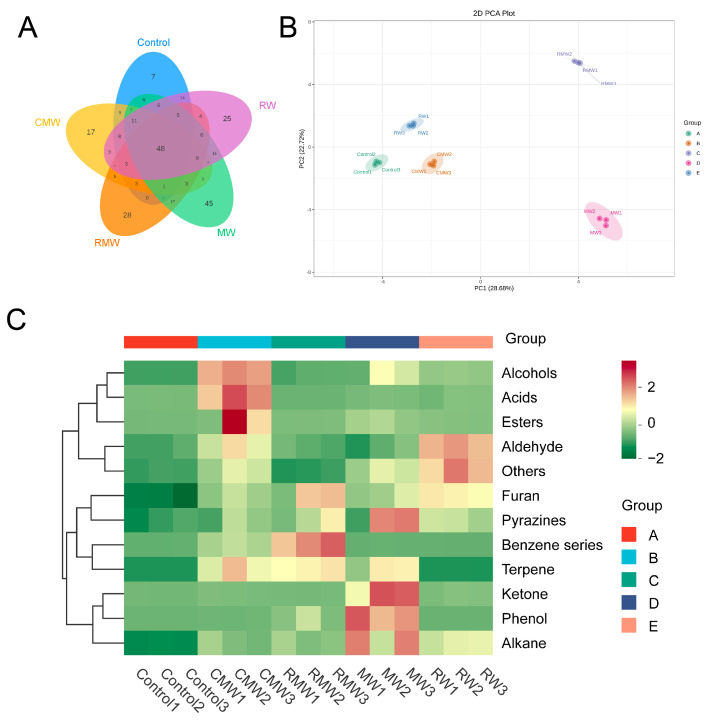
(**A**) Venn diagram of the groups of volatile compounds in chicken wings before and after smoking with four spices. (**B**) PCA scores plot based on the concentration of the volatile compounds in chicken wings before and after smoking with four spices. (**C**) Cluster heat map based on the concentrations of different groups of volatile compounds in chicken wings before and after smoking with four spices.

Figure 3A shows the Venn diagram illustrating the distribution of volatile compound types in chicken wings smoked with four kinds of spices. A total of 48 volatile compounds were common across all five samples, while the control sample contained the fewest unique compounds (*n* = 7). In contrast, the number of unique volatiles increased markedly in the spice-smoked samples, with 45, 28, 25, and 17 unique compounds detected in the MW, RMW, RW, and CMW samples, respectively. These findings suggest that the smoking process substantially enhances the diversity of volatile compounds in the chicken wings. To further explore the differences in volatile profiles, PCA was performed based on the concentration data of all detected volatiles. As shown in Figure 3B, the first two principal components account for 51.4% of the total variation. The PCA plot demonstrates a clear separation among all samples, indicating notable differences in flavor composition due to spice smoking. Notably, CMW, RW, and control samples were positioned closer together in the PCA space, whereas MW and RMW were located further from the control, suggesting that mint and rosemary smoking exerted a more pronounced impact on the flavor profile of the chicken wings.

A hierarchical clustering heatmap further illustrated the similarities and differences in the profiles of high-abundance volatile compounds across the samples (Figure 3C). The control sample exhibited generally low abundance across all compound categories, whereas distinct enrichment patterns were observed in the spice-smoked samples. Specifically, esters, acids, and alcohols were most abundant in CMW; benzene series, furans, and terpenes were dominant in RMW; MW displayed higher enrichment in ketones, phenols, alkanes, and pyrazines; and RW showed high levels of aldehydes, furans, and others. In summary, the analyses reveal that smoking with different spices significantly enhances the types and concentration of aroma volatiles in chicken wings. Furthermore, each spice contributed characteristic volatiles reflective of its own aroma profile, which were effectively transferred and enriched in the smoked chicken wings.

### 3.3. Metabolomic Analysis of Differential Volatiles

To obtain a more comprehensive understanding of the metabolite composition profiles, differentially accumulated metabolites (DAMs) were identified based on two criteria: variable importance of the projection (VIP) ≥ 1 and |log_2_(fold change)| ≥ 1. The VIP value was analyzed based on the OPLSR. Anal function of the MetaboAnalystR package. Fold change (FC) was calculated as the ratio of metabolite abundance in the experimental group relative to the control group. Following data normalization, an OPLS-DA model was constructed, which revealed robust separation among five sample groups. Model validation and permutation test parameters for each comparison group are shown in Table S2. All models demonstrated strong statistical performance, with R^2^X values exceeding 0.821, R^2^Y values equal to 1, and Q^2^ values greater than 0.98, indicating excellent explanatory power for both X and Y matrices as well as high predictive reliability. The OPLS-DA score plots for all comparison groups are shown in Figure S1, which illustrates clear group differentiation. These results confirm that chicken wings smoked with different spices exhibited significant differences in metabolite profiles compared to unsmoked controls. Differential metabolites were further screened using a combination of fold change analysis, *t*-tests, and VIP scores from the OPLS-DA models. Metabolites meeting the thresholds of |log_2_FC| ≥ 1, *p* < 0.05, and VIP > 1 were designated as differential volatiles.

**Figure 4:**
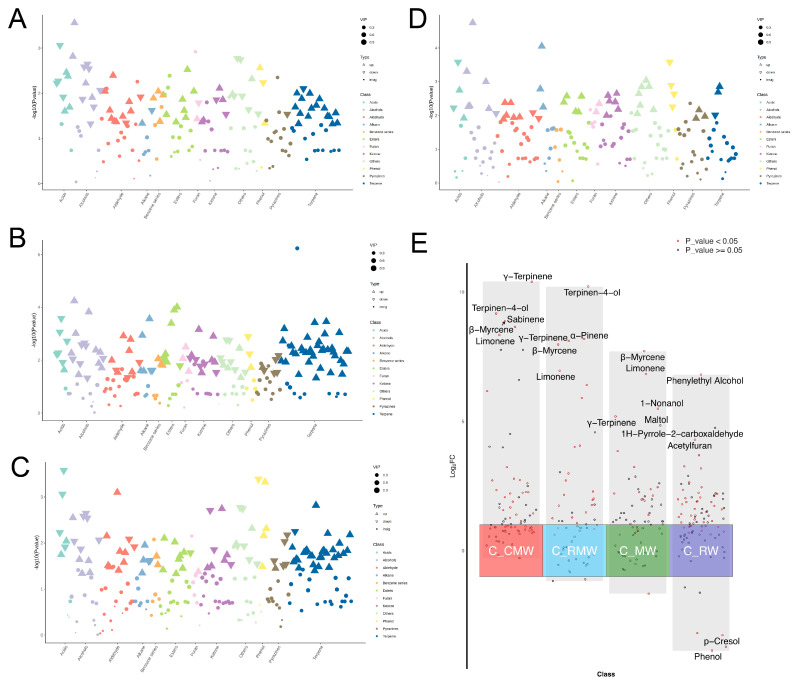
(**A**–**D**) Scatter plot of differential volatiles classification. (**A**–**D**) are the comparison groups of chicken wings smoked with cardamom, rosemary, mint, and rose, respectively, and those smoked without spices. In the figure, the upright triangles represent the up-regulated compounds, the inverted triangles represent the down-regulated compounds, and the circles represent the compounds with no significant difference; the size of the triangle/circle represents the VIP value. (**E**) Multi-group differential volcano map. The coordinates on the central axis from left to right are the comparison groups of chicken wings smoked with cardamom, rosemary, mint, and rose, respectively, and those smoked without spices. The compounds labeled are the top 3 log_2_FC compounds in each comparison group.

Scatter plots illustrating the classification of differential volatiles across the four comparison groups (cardamom, rosemary, mint, and rose) are shown in Figure 4A–D. The number of significantly upregulated compounds in the respective groups were 54, 79, 69, and 42, while the number of downregulated compounds were 26, 39, 31, and 10, respectively. In all cases, the number of upregulated compounds was significantly higher than that of downregulated compounds. Among these, terpenes represented the most significantly upregulated class following smoking with cardamom, rosemary, and mint, with 15, 30, and 26 terpenes upregulated, respectively. In contrast, rose-smoked chicken wings showed the most prominent increases in aldehydes (6), alcohols (6), phenols (6), and miscellaneous volatiles (7). A multi-group differential volcano map (Figure 4E) shows the top five compounds in each comparison group based on |log_2_FC|. In the C_CMW and C_RMW groups, the top five compounds were all terpenes, including terpinen-4-ol, γ-terpinene, β-myrcene, and limonene. Notably, C_CMW and C_MW both had β-myrcene and limonene. The compound with the highest |log_2_FC| in C_RW is phenylethyl alcohol, which is the compound with a typical rose aroma. Overall, most of the differential volatiles in different comparison groups were significantly up-regulated after spice smoking, which further proved that the smoking process had a significant aroma-imparting effect on chicken wings.

### 3.4. ROAV Analysis of Volatiles in Four Spice-Smoked Chicken Wings

The relative odor activity value (ROAV) is defined as the ratio of a compound’s relative concentration to its odor threshold in water [20]. Compounds with ROAV ≥ 1 were deemed to contribute to the aroma profile of the sample [21]. Table 1 lists the 45 volatiles with ROAV ≥ 1 across the five samples. Among these, eucalyptol exhibited the highest ROAV, ranging from 4 to 2543, and was associated with minty, camphoraceous, and eucalyptus-like sensory notes. In the control sample, compounds with the highest ROAV values were primarily aldehydes, including isovaleraldehyde (ROAV = 169; chocolate, fatty), 2-methylbutanal (ROAV = 104; musty, chocolate, nutty), isobutyraldehyde (ROAV = 57; fresh, floral, green), and (*E*,*E*)-2,4-decadienal (ROAV = 39; fatty, chicken, fried). These compounds also corresponded to volatiles with relatively high concentrations in the control group. Notably, the volatile profiles of samples smoked with cardamom and rosemary (CMW and RMW) were highly similar, both characterized by dominant terpenes and aldehydes with elevated ROAV values. In the CMW sample, major contributors included eucalyptol (ROAV = 2543), β-myrcene (ROAV = 329), linalool (ROAV = 250), isovaleraldehyde (ROAV = 194), and (*E*,*E*)-2,4-decadienal (ROAV = 184). Similarly, in the RMW sample, eucalyptol (ROAV = 2115), linalool (ROAV = 928), β-myrcene (ROAV = 175), isovaleraldehyde (ROAV = 141), and 2-methylbutanal (ROAV = 133) were the most prominent contributors. In the MW group, terpenes and aldehydes also predominated, though some non-terpene compounds showed high ROAVs as well. For instance, 2,3-butanedione (ROAV = 127; sweet, creamy, caramel-like), methanethiol (ROAV = 53; vegetable, alliaceous), and ethyl α-methylbutyrate (ROAV = 52; fruity, estery, berry-like) were notable contributors. In the RW sample, aldehydes again dominated the list of key odorants, with 2-methylbutanal (ROAV = 389) showing the highest ROAV, followed by isovaleraldehyde (ROAV = 259), isobutyraldehyde (ROAV = 117), and dimethyl sulfide (ROAV = 52). Importantly, there was no straightforward positive correlation between compound concentration and ROAV. For example, although 2-methylbutanal and isovaleraldehyde were not among the most abundant compounds, their extremely low odor thresholds (0.001 mg/kg and 0.0011 mg/kg, respectively) resulted in exceptionally high ROAVs, making them significant aroma contributors. In contrast, ethanol, present at high concentrations (275.48–5559.78 μg/kg), had a much higher threshold (950 mg/kg) and thus contributed minimally to the perceived aroma. Generally, the combination of quantitative analysis and ROAV calculation can describe the aroma profiles more accurately.

To further assess the impact of spice smoking, ROAV changes were calculated by comparing samples smoked with spices to the control group. Interestingly, the results differed from ROAV distributions observed within individual samples. In the MW sample, most of the compounds with the largest ROAV increases were aldehydes. However, in the other three spice-smoked groups (CMW, RMW, and RW), the compounds with the greatest ROAV increases were primarily terpenes and alcohols. This finding suggests that the majority of aldehydes may not originate from the spices themselves but rather are characteristic products of thermal reactions within the meat matrix during the smoking process [10,22].

### 3.5. Analysis of Key Aroma Profile of Smoked Chicken Wings

Based on the above analysis, key aroma compounds were identified by integrating volatiles that met the following criteria: |log_2_FC| ≥ 1, *p* < 0.05, VIP > 1, and △ROAV (the difference in ROAV between compounds with and without spices) > 1 across the four comparison groups. These compounds were considered representative of the characteristic aroma contributions of each spice in smoked chicken wings. A Venn diagram (Figure S2) was constructed to visualize the distribution of key aroma compounds identified in the comparison groups of cardamom (CMW), rosemary (RMW), mint (MW), and rose (RW) against the control. A total of 24 volatiles were identified across all groups. Specifically, 7, 13, 10, and 4 key volatiles were identified in CMW, RMW, MW, and RW, respectively. The number of unique compounds for each group was 2 (CMW), 8 (RMW), 4 (MW), and 3 (RW), respectively. Eucalyptol emerged as a common key aroma compound across all four spice-smoked groups. β-Myrcene was shared among the CMW, RMW, and MW groups. Methanethiol and α-pinene were common to RMW and MW, while benzeneacetaldehyde and limonene were shared between CMW and RMW and CMW and MW, respectively. These findings suggest distinct aroma profiles and compound specificity among the different spices, highlighting the critical role of spice selection in the smoking process. The above results highlight the volatile compounds released from different spices during the smoking process and their subsequent transfer into chicken wings. Following spice smoking, the key aroma compounds detected in the chicken wings were predominantly terpenes, which are commonly recognized as characteristic volatile constituents naturally present in spices. The widespread occurrence of eucalyptol and β-myrcene suggests that these compounds possess high thermal stability and volatility, as well as a strong affinity for adsorption onto the chicken wing matrix. In contrast, variations in the presence of compounds such as methanethiol, α-pinene, phenylacetaldehyde, and limonene reflect the distinct chemical compositions of individual spices and their divergent behaviors under thermal processing. For key aroma compounds exhibiting favorable thermal stability, high volatility, and strong adsorption capacity, future studies will investigate their adsorption kinetics and optimize the adsorption process. These efforts aim to provide a mechanistic understanding of the release and adsorption behavior of key aroma compounds during the smoking process.

**Figure 5:**
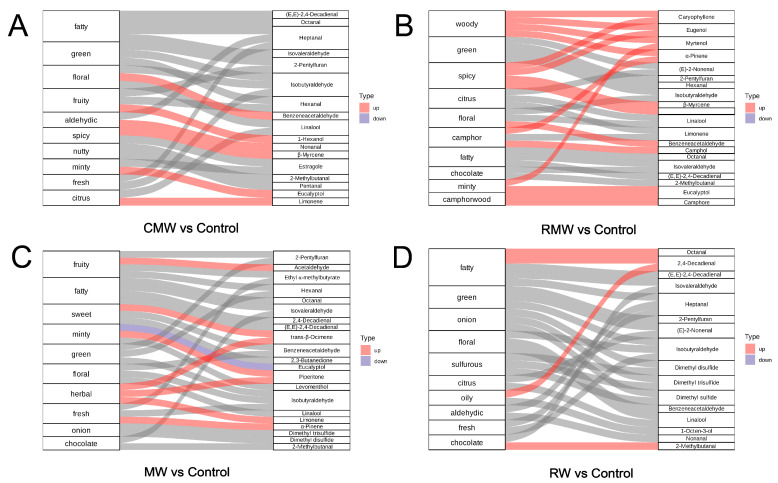
(**A**–**D**) Sankey diagrams of key differential volatile compounds in smoked chicken wings with different spices; (**A**–**D**) are the comparison groups of chicken wings smoked with cardamom, rosemary, mint, and rose, respectively, and those smoked without spices. The left is the sensory feature, the right is the differential volatiles with △ROAV > 1, the red flow line represents the significantly up-regulated flow of volatiles, and the blue flow line represents the significantly down-regulated flow of volatiles, the gray flow line represents the up-regulation/down-regulation results are not statistically significant.

To further visualize the impact of key differential volatiles on sensory characteristics, Sankey diagrams were generated for each comparison group (Figure 5A–D), illustrating how changes in volatile composition influenced the main odor attributes. Across all four comparison groups, a variety of odor notes were altered following spice smoking, with most being significantly enhanced. In the diagrams, red and blue streamlines represent the key differential volatiles; the predominance of upward shifts indicates that these compounds largely contributed to the intensification of aroma attributes. In chicken wings smoked with cardamom (Figure 4A), the most prominent changes were observed in fatty (4 volatiles), green (3), floral (3), and fruity (3) odor attributes, with floral and fruity notes showing the greatest enhancement. In the rosemary group (Figure 5B), the key changes were in woody, green, and spicy notes (4 volatiles each), with all contributing volatiles enhancing their respective attributes. Notably, minty and camphoraceous characteristics were also significantly intensified. For the mint-smoked wings (Figure 5C), the herbal note was significantly enhanced, driven by three upregulated volatiles, alongside notable increases in fruity, sweet, minty, and fresh notes. In the rose-smoked sample (Figure 5D), fatty odor characteristics were most affected, with five volatiles involved. Two of these, octanal and 2,4-decadienal, were significantly upregulated, contributing strongly to the fatty profile. The spice-smoking treatments resulted in distinct aroma profiles that significantly influenced the sensory characteristics of smoked chicken wings. These differences are likely to affect consumer preferences, with fruity, sweet, and herbal aromas typically associated with freshness and higher consumer acceptance, particularly among younger or aroma-sensitive groups [23]. In contrast, woody and fatty notes may appeal to consumers seeking traditional or rich meat flavors [24]. The presence of thermally stable, high-affinity compounds like eucalyptol and β-myrcene indicates their strong potential for consistent aroma delivery. These findings suggest that spice selection can be strategically used to tailor flavor profiles in line with market demands. Optimizing the release and adsorption of key volatiles—through controlled smoking conditions and spice combinations—offers a pathway for precise flavor modulation. Such insights can be extended beyond chicken wings to other smoked meat products, where targeted aroma enhancement can improve sensory appeal or mask undesirable notes. Future studies should explore the adsorption kinetics of key volatiles to further refine aroma retention and stability.

Our findings indicate that rose-smoked chicken wings exhibited enhanced fatty, oily, and chocolate-like odor characteristics, primarily attributed to an accumulation of various aldehydes. However, it is important to note that the perceived odor of a compound can vary depending on its concentration, the substrate matrix, and its interaction with other volatiles [25]. For example, aldehydes can show strong odor characteristics such as bitter, chocolate, and greasy and can also exhibit a variety of odor characteristics such as green, woody, honey, and floral [26]. Therefore, in our sample, their formation is likely to be partly related to lipid oxidation and Strecker degradation, mainly showing fatty odor, or it may be transferred from rose release, mainly showing floral odor [27,28]. In the future, GC-O and other technologies are needed to further analyze the true odor contribution of different aldehydes. In general, floral, green, fruity, spicy, fresh, fatty, and other odor characteristics of chicken wings smoked with different spices were enhanced to varying degrees, which was consistent with our preliminary sensory evaluation results on smoked chicken wings, indicating that smoking chicken wings with spices is an effective way to add fragrance. Based on these results, the smoking process may be further optimized and tailored according to ingredient characteristics, spice combinations, and consumer preference profiles.

## 4. Conclusions

This study integrates traditional smoking concepts with modern analytical techniques to elucidate the aroma-enhancing potential of spices in home-smoked chicken wings. The combined use of air frying and a portable griddle-based smoking system effectively minimized lipid oxidation and potential carcinogen formation while enabling efficient transfer of spice-derived volatiles. Spice smoking significantly increased the diversity and concentration of volatiles in chicken wings, with terpenes (e.g., eucalyptol) and aldehydes emerging as predominant contributors. Of the 45 odor-active compounds identified (ROAV ≥ 1), eucalyptol exhibited the highest ROAV (2543), primarily contributing minty and woody sensory notes. Metabolomic analysis revealed spice-specific modulation of volatiles: terpenes were notably upregulated in cardamom-, rosemary-, and mint-smoked samples, while aldehydes and alcohols were predominant in rose-smoked wings. Integrated screening identified 24 key volatiles (e.g., β-myrcene and α-pinene), defining distinct aroma profiles. These findings not only validate the feasibility of spice smoking in chicken wing flavor processing but also provide a scientific basis for optimizing flavor formulations in smoked meat products and other foods. Future research could explore synergistic spice blends and their interactions with meat matrices under varying thermal conditions. By harmonizing tradition with innovation, this work offers a scientific foundation for flavor customization in both household and industrial contexts.

## Figures and Tables

**Table 1:** ROAV (≥1) of volatile flavor compounds of chicken wings before and after smoking with four spices.

Compounds	Odor Threshold (mg/kg) ^a,b^	Aroma Description ^c^	ROAV ^d^				
			Control ^e^	CMW ^e^	RMW ^e^	MW ^e^	RW ^e^
Benzene series							
*p*-Cymene	0.00501	mild, pleasant	n.d	88	n.d	n.d	n.d
β-Cymene	0.8	/	n.d	n.d	2	n.d	n.d
*p*-Cymenene	0.085	spicy	n.d	n.d	1	<1	n.d
Pyrazines							
2-Ethyl-3,6-dimethylpyrazine	0.0086	potato, cocoa, roasted	3	5	5	6	4
Alcohols							
1-Octen-3-ol	0.0015	mushroom, earthy, oily	8	23	52	8	13
1-Hexanol	0.0056	pungent, fruity, alcoholic	1	3	<1	<1	<1
Isopentyl alcohol	0.004	fusel, alcoholic, pungent	<1	n.d	n.d	n.d	2
Methanethiol	0.0002	vegetable, alliaceous	n.d	n.d	53	53	31
3-Octanol	0.078	mushroom, dairy	n.d	n.d	<1	4	n.d
Furan							
2-Pentylfuran	0.0058	fruity, green, earthy beany	2	12	15	8	10
Aldehyde							
Isovaleraldehyde	0.0011	chocolate, fatty	169	194	141	111	259
Hexanal	0.005	green, vegetative, fruity	24	62	14	18	25
2-Methylbutanal	0.001	musty, chocolate, nutty	104	172	133	101	389
Isobutyraldehyde	0.0015	fresh, floral, green	57	108	51	54	117
Benzeneacetaldehyde	0.0063	honey, floral, sweet	4	12	9	9	12
Pentanal	0.012	fermented, bready, nutty	2	3	1	1	1
Nonanal	0.0011	waxy, aldehydic	14	46	27	29	31
Heptanal	0.0028	fresh, aldehydic, fatty	3	6	3	3	5
Octanal	0.000587	fatty	10	57	19	17	22
(*E*,*E*)-2,4-Decadienal	0.000027	fatty, chicken, fried	39	184	n.d	n.d	n.d
(*E*)-2-Nonenal	0.00019	green, cucumber, citrus	n.d	n.d	15	n.d	7
Acetaldehyde	0.0251	pungent, aldehydic, fruity	n.d	n.d	n.d	2	n.d
2,4-Decadienal	0.0003	fatty, oily, chicken skin-like	n.d	n.d	n.d	18	12
Terpene							
Linalool	0.00022	citrus, floral, rose	11	250	928	158	14
Eucalyptol	0.0011	minty, camphorwood, eucalyptus	35	2543	2115	11	4
Limonene	0.2	citrus, herbal, camphor	<1	10	4	4	<1
β-Myrcene	0.0012	pepper, spicy	<1	329	175	146	3
α-Pinene	0.014	fresh, camphor, earthy, woody	<1	n.d	41	3	<1
α-Terpinene	0.08	woody, lemon, medicinal	<1	1	<1	n.d	n.d
*trans*-β-Ocimene	0.034	herbal, sweet	n.d	<1	<1	7	n.d
Camphore	0.52	camphorwood	n.d	<1	2	n.d	n.d
Caryophyllene	0.064	spicy, woody	n.d	<1	3	<1	n.d
Camphol	0.18	wood, camphor	n.d	n.d	8	n.d	n.d
Levomenthol	2.28	minty	n.d	n.d	<1	1	n.d
Myrtenol	0.007	woody, pine, minty	n.d	n.d	2	n.d	n.d
Eugenol	0.00071	spicy, clove, woody	n.d	n.d	2	<1	n.d
Piperitone	0.68	herbal, minty, camphorwood	n.d	n.d	n.d	1	n.d
Ketone							
Acetoin	0.014	sweet, buttery, creamy	<1	n.d	<1	<1	1
2,3-Butanedione	0.000059	sweet, creamy, caramellike	n.d	n.d	n.d	127	n.d
Esters							
Ethyl acetate	0.005	fruity, sweet, grape	<1	1	<1	<1	1
Ethyl α-methylbutyrate	0.000007	fruity, estery, berry, fresh	n.d	n.d	n.d	52	n.d
Others							
Dimethyl disulfide	0.0011	sulfurous, cabbage, onion	4	5	2	2	n.d
Dimethyl trisulfide	0.0001	sulfurous, onion, meaty	10	23	n.d	n.d	n.d
Estragole	0.006	phenolic, anise, spicy, minty	n.d	2	n.d	n.d	<1
Dimethyl sulfide	0.00012	sulfurous, onion, corn, cabbage	n.d	n.d	n.d	26	52

^a^ Odor threshold referred to the value detected in water. ^b^ Odor thresholds were from reference Van Gemert, L. J. [18]. ^c^ The amora description refers to the information recorded in https://www.perflavory.com/search.php (accessed on 28 January 2025). ^d^ ROAV was equal to the relative odor concentration divided by the threshold. ^e^ Control represented smoked chicken wings without spices; CMW, RMW, MW, and RW represented smoked chicken wings with cardamom, rosemary, mint, and rose, respectively. n.d, not detected.

## Data Availability

This research received no external funding. The original contributions presented in the study are included in the article, further inquiries can be directed to the corresponding author.

## Supplementary Materials

The following supporting information can be downloaded at: https://www.mdpi.com/article/10.3390/foods14132270/s1, Figure S1: OPLS-DA analysis results of volatile compounds in chicken wings before and after smoking with four spices. Figures A, C, E, and G are the two-dimensional scores plot of cardamom, rosemary, mint, and rose, respectively. Figures B, D, F, and H are the S-plot of cardamom, rosemary, mint, and rose, respectively; Figure S2: Venn diagram of key differential volatile compounds in smoked chicken wings with different spices. The bar graph shows the number of key volatile compounds after smoked chicken wings with each spice; Table S1: Identification and quantitation of volatile flavor compounds in chicken wings before and after smoking with four spices’ Table S2: Permutation test parameters of OPLS-DA models.
