# Supplementary material for: Metabolomic Insights into Volatile Profiles and Flavor Enhancement of Spice-Smoked Chicken Wings
Source: Foods. 2025 Jun 26;14(13):2270. doi: 10.3390/foods14132270 (PMC12248852; doi:10.3390/foods14132270)

Table S1. Identification and quantitation of volatile flavor compounds in chicken wings before and after smoking with four spices.

Compounds	CAS	Retention Index	Concentration (μg/kg)				
			Control [e]	CMW [e]	RMW [e]	MW [e]	RW [e]
**Benzene series**							
Toluene	108-88-3	1032.6	10.90±2.95[bc]	34.64±6.54[a]	7.88±0.19[c]	8.63±2.55[c]	16.19±2.76[b]
o-Cymene	527-84-4	1262.4	1.83±2.20[b]	n.d[b]	n.d[b]	10.02±1.54[a]	2.00±0.87[b]
o-Xylene	95-47-6	1128.2	0.46±0.32[b]	1.93±0.11[a]	0.79±0.07[b]	n.d[c]	0.89±0.38[b]
p-Cymene	99-87-6	1262.2	n.d[b]	438.70±80.30[a]	n.d[b]	n.d[b]	n.d[b]
Styrene	100-42-5	1248.9	n.d[b]	2.27±0.92[ab]	n.d[b]	3.44±2.98[a]	4.82±1.40[a]
p-Xylene	106-42-3	1121.7	n.d[c]	1.53±0.56[a]	n.d[c]	0.83±0.25[b]	1.37±0.39[ab]
Ethylbenzene	100-41-4	1115	0.71±0.07[a]	1.34±0.19[a]	0.65±0.10[a]	0.61±0.07[a]	1.38±1.26[a]
β-Cymene	535-77-3	1264.3	n.d[b]	n.d[b]	1576.14±328.62[a]	n.d[b]	n.d[b]
p-Cymenene	1195-32-0	1431	n.d[b]	n.d[b]	119.66±19.68[a]	7.83±1.91[b]	n.d[b]
m-Ethyltoluene	620-14-4	1372.4	n.d[b]	n.d[b]	1.65±0.36[a]	n.d[b]	n.d[b]
**Pyrazines**							
2,5-Dimethylpyrazine	123-32-0	1318.2	39.06±10.42[b]	63.29±4.63[ab]	86.16±39.37[ab]	130.35±74.41[a]	82.98±16.74[ab]
Trimethylpyrazine	14667-55-1	1401.5	28.66±7.25[b]	41.73±7.90[ab]	57.91±26.79[ab]	93.70±57.02[a]	54.01±10.91[ab]
2-Ethyl-3,6-dimethylpyrazine	13360-65-1	1444.4	25.14±6.41[b]	41.17±11.27[ab]	45.84±5.17[ab]	55.23±24.28[a]	35.26±11.50[ab]
Methylpyrazine	109-08-0	1260.9	24.06±6.16[b]	44.97±39.68[ab]	66.44±20.14[ab]	82.52±35.37[a]	39.58±23.08[ab]
2,6-Dimethylpyrazine	108-50-9	1324.6	16.90±5.56[a]	31.67±4.83[a]	31.32±7.86[a]	33.84±18.85[a]	25.97±9.25[a]
2-Ethyl-6-methylpyrazine	13925-03-6	1382.8	11.59±3.93[b]	19.88±4.46[ab]	21.29±7.11[ab]	30.10±7.78[a]	16.33±14.16[ab]
2,3-Dimethylpyrazine	5910-89-4	1342	7.83±1.98[b]	11.45±1.52[ab]	11.99±1.51[ab]	14.66±6.81[a]	13.36±1.98[ab]
Ethylpyrazine	13925-00-3	1330.2	2.08±1.90[b]	10.56±1.59[a]	10.16±0.26[a]	14.27±6.25[a]	13.02±2.44[a]
2-Methyl-3,5-diethylpyrazine	18138-05-1	1494	2.90±0.54[a]	6.05±1.95[a]	5.58±3.39[a]	2.55±1.09[a]	5.57±1.84[a]

Pyrazine	290-37-9	1206.2	1.55±1.35^{ab}	n.d^{b}	n.d^{b}	3.39±3.51^{ab}	3.65±1.52^{a}
2,5-Dimethyl-3-isobutylpyrazine	32736-94-0	1526.1	1.92± 0.32^{bc}	4.38±2.23^{a}	n.d^{c}	n.d^{c}	2.37±0.97^{b}
2,6-Diethylpyrazine	13067-27-1	1432.5	1.29±0.39^{b}	n.d^{c}	n.d^{c}	n.d^{c}	2.85±0.25^{a}
2-Methyl-6-vinylpyrazine	13925-09-2	1485.6	0.96±0.30^{c}	2.25±0.67^{b}	n.d^{d}	n.d^{d}	3.32 ±0.64^{a}
2,3,5-Trimethyl-6-ethylpyrazine	17398-16-2	1514.2	0.95±0.13^{b}	1.77±0.87^{a}	n.d^{c}	n.d^{c}	0.91±0.16^{b}
2-Methyl-5-isopropylpyrazine	13925-05-8	1408.8	0.15±0.10^{a}	n.d^{b}	n.d^{b}	n.d^{b}	0.21±0.02^{a}
2-Ethyl-3,5-dimethylpyrazine	13925-07-0	1460.9	11.77±3.24^{b}	9.64±2.48^{b}	n.d^{c}	n.d^{c}	22.34±3.12^{a}
2-Ethyl-5-methylpyrazine	13360-64-0	1388.2	n.d^{b}	n.d^{b}	29.29±7.11^{ab}	37.13±32.15^{a}	36.62±12.82^{a}
Acetylpyrazine	22047-25-2	1620.4	n.d^{b}	n.d^{b}	n.d^{b}	n.d^{b}	1.14±1.05^{a}
5H-5-Methyl-6,7-dihydrocyclopentapyrazine	23747-48-0	1618.8	n.d^{b}	n.d^{b}	n.d^{b}	n.d^{b}	0.75±0.23^{a}
2-Propylpyrazine	18138-03-9	1414.5	n.d^{b}	n.d^{b}	n.d^{b}	n.d^{b}	0.44±0.08^{a}
**Alcohols**							
1-Octen-3-ol	3391-86-4	1452.3	11.77±3.24^{c}	35.21±10.96^{b}	77.29±4.35^{a}	11.41±2.41^{c}	19.57±2.07^{c}
1-Hexanol	111-27-3	1355.3	6.46±1.90^{b}	17.55±2.69^{a}	4.41±2.11^{b}	3.85±0.39^{b}	5.33±2.06^{b}
2,3-Butanediol	513-85-9	1577.3	5.74±0.96^{b}	6.07±5.28^{b}	n.d^{c}	n.d^{c}	24.86±3.11^{a}
1-Pentanol	71-41-0	1252.9	4.60±1.04^{b}	8.09±2.15^{b}	6.68±1.16^{b}	7.04±0.47^{b}	11.78±3.20^{a}
1-Butanol	71-36-3	1148.6	2.48±2.15^{a}	3.20±2.78^{a}	1.10±0.44^{a}	1.66±1.17^{a}	4.01±0.54^{a}
2-Furanmethanol	98-00-0	1659	3.63±0.68^{d}	6.22±1.42^{c}	8.83±0.76^{b}	3.12±0.98^{d}	16.64±0.61^{a}
Isopentyl alcohol	123-51-3	1210.3	3.33±0.68^{b}	n.d^{c}	n.d^{c}	n.d^{c}	8.49±3.38^{a}
1-Penten-3-ol	616-25-1	1163.2	1.97±0.49^{ab}	n.d^{c}	1.57±0.91^{b}	2.25±0.39^{ab}	2.65±0.28^{a}
1-Heptanol	111-70-6	1457.8	1.87±0.30^{a}	n.d^{b}	n.d^{b}	n.d^{b}	n.d^{b}
Phenylethyl Alcohol	60-12-8	1909	1.58±0.47^{b}	6.36±0.50^{b}	10.13±1.45^{b}	9.47±1.16^{b}	176.48±22.46^{a}
1-Propanol	71-23-8	1040.6	1.54±0.13^{b}	n.d^{c}	n.d^{c}	n.d^{c}	2.59±1.10^{a}
2-Ethyl-1-hexanol	104-76-7	1491.6	1.27±0.11^{ab}	n.d^{b}	n.d^{b}	n.d^{b}	3.98±3.45^{a}

Isobutyl alcohol	78-83-1	1099.5	0.57±0.51[b]	0.95±0.83[ab]	0.58±0.18[b]	0.56±0.22[b]	1.55±0.45[a]
1-Nonanol	143-08-8	1662.7	0.16±0.06[b]	n.d[b]	n.d[b]	7.37±1.99[a]	0.28±0.32[b]
5-Methyl-2-furfuryl alcohol	3857-25-8	1720.9	0.09±0.01[b]	n.d[b]	n.d[b]	n.d[b]	0.53±0.25[a]
Ethanol	64-17-5	931.8	n.d[c]	5559.78±376.27[a]	275.48±238.71[c]	1902.85±1418.61[b]	1240.79±47.79[bc]
(*Z*)-2,3-butanediol	24347-58-8	1577.5	n.d[b]	6.69±5.81[a]	n.d[b]	n.d[b]	n.d[b]
3-(Methylthio)-1-propanol	505-10-2	1715.9	n.d[c]	0.29±0.06[a]	n.d[c]	n.d[c]	0.17±0.02[b]
p-Cymen-8-ol	1197-01-9	1847.9	n.d[b]	n.d[b]	36.24±4.05[a]	n.d[b]	n.d[b]
Methanethiol	74-93-1	672.3	n.d[b]	n.d[b]	10.69±3.58[a]	10.64±2.83[a]	6.28±5.44[a]
3-Octanol	589-98-0	1396	n.d[b]	n.d[b]	4.31±0.09[b]	334.41±125.52[a]	n.d[b]
1-Decanol	112-30-1	1764.1	n.d[b]	n.d[b]	2.65±2.31[ab]	4.84±2.07[a]	n.d[b]
1-Octanol	111-87-5	1560.1	n.d[b]	n.d[b]	3.88±2.13[a]	n.d[b]	n.d[b]
1-Undecanol	112-42-5	1866.3	n.d[b]	n.d[b]	0.84±0.17[a]	n.d[b]	n.d[b]
(*E*)-2-Penten-1-ol	1576-96-1	1312.9	n.d[b]	n.d[b]	0.31±0.04[a]	n.d[b]	n.d[b]
Limonene glycol	1946-00-5	2271.5	n.d[b]	n.d[b]	0.20±0.05[a]	n.d[b]	n.d[b]
(2S,3S)-(+)-2,3-Butanediol	19132-06-0	1539.9	n.d[b]	n.d[b]	n.d[b]	3.94±3.49[a]	n.d[b]
Benzyl alcohol	100-51-6	1872.8	n.d[c]	n.d[c]	n.d[c]	2.34±0.90[b]	4.31±1.13[a]
3-Hexen-1-ol	544-12-7	1384.4	n.d[b]	n.d[b]	n.d[b]	1.13±1.06[a]	n.d[b]
1,2-Ethanediol	107-21-1	1623	n.d[b]	n.d[b]	n.d[b]	n.d[b]	0.53±0.03[a]
1,4-Butanediol	110-63-4	1920.9	n.d[b]	n.d[b]	n.d[b]	n.d[b]	0.13±0.11[a]
**Phenol**							
Phenol	108-95-2	2000.9	0.33±0.01[c]	0.93±0.06[a]	0.65±0.21[b]	n.d[d]	0.68±0.08[b]
Maltol	118-71-8	1969.6	0.28±0.02[b]	0.54±0.47[b]	n.d[b]	8.16±7.75[a]	n.d[b]
o-Guaiacol	90-05-1	1854.4	0.12±0.02[ab]	n.d[b]	n.d[b]	0.49±0.47[a]	0.39±0.10[ab]
p-Cresol	106-44-5	2077.2	0.08±0.01[b]	0.34±0.10[b]	1.34±0.30[a]	0.35±0.09[b]	0.18±0.05[b]
Thymol	89-83-8	2182.1	n.d[b]	n.d[b]	10.42±13.87[b]	73.03±10.57[a]	n.d[b]

Methyleugenol	93-15-2	2009.3	n.d[b]	n.d[b]	2.10±0.92[a]	n.d[b]	0.22±0.06[b]
2-Methoxy-4-vinylphenol	7786-61-0	2189.6	n.d[b]	n.d[b]	0.81±0.54[a]	0.59±0.02[a]	n.d[b]
Diosphenol	490-03-9	1805.2	n.d[b]	n.d[b]	n.d[b]	5.50±0.67[a]	n.d[b]
2,4-Di-tert-butylphenol	96-76-4	2311	n.d[b]	n.d[b]	n.d[b]	n.d[b]	0.19±0.11[a]
**Furan**							
2-Pentylfuran	3777-69-3	1226.8	10.52±9.12[c]	69.27±8.15[ab]	86.20±39.00[a]	46.06±7.14[b]	57.62±3.25[ab]
2-Ethylfuran	3208-16-0	943.8	6.11±1.37[b]	n.d[d]	6.64±1.02[b]	4.29±0.53[c]	9.90±1.08[a]
Acetylfuran	1192-62-7	1498.9	2.11±0.30[b]	4.95±1.08[b]	8.51±1.21[b]	9.44±3.61[b]	41.15±9.07[a]
2-n-Butyl furan	4466-24-4	1124.8	0.59±0.51[a]	2.07±1.85[a]	2.03±0.75[a]	0.90±0.80[a]	2.34±0.23[a]
2-Methylfuran	534-22-5	862.5	n.d[c]	n.d[c]	2.54±0.25[a]	1.42±0.52[b]	n.d[c]
2-Vinylfuran	1487-18-9	1068.9	n.d[b]	n.d[b]	1.21±0.35[a]	n.d[b]	n.d[b]
Menthofuran	494-90-6	1478.2	n.d[b]	n.d[b]	n.d[b]	15.85±6.39[a]	n.d[b]
2-Hexylfuran	3777-70-6	1226.8	n.d[b]	n.d[b]	n.d[b]	1.84±1.68[a]	n.d[b]
**Aldehyde**							
Isovaleraldehyde	590-86-3	912.3	185.51±20.19[ab]	213.44±36.82[ab]	154.96±17.87[b]	122.45±41.76[b]	284.47±115.45[a]
Hexanal	66-25-1	1076.2	120.81±36.68[b]	310.49±137.02[a]	68.00±10.70[b]	92.47±28.02[b]	124.49±56.94[b]
2-Methylbutanal	96-17-3	909	104.25±13.11[c]	172.34±28.44[b]	133.35±23.31[bc]	101.37±43.76[c]	388.63±51.87[a]
Isobutyraldehyde	78-84-2	809.6	85.06±16.80[b]	161.69±6.91[a]	76.83±3.87[b]	80.50±25.90[b]	176.58±39.36[a]
Benzeneacetaldehyde	122-78-1	1633.7	24.88±5.59[b]	73.86±15.97[a]	59.29±9.49[a]	56.97±19.61[a]	76.15±14.46[a]
Pentanal	110-62-3	970.3	18.54±3.32[b]	37.66±12.70[a]	12.51±3.59[b]	16.28±3.85[b]	15.68±8.78[b]
Nonanal	124-19-6	1389.5	15.92±2.54[c]	50.69±12.50[a]	29.34±6.61[bc]	32.15±4.04[b]	33.87±11.84[b]
Benzaldehyde	100-52-7	1515	10.69±2.92[c]	28.11±7.43[b]	n.d[c]	40.82±10.82[a]	27.41±3.68[b]
Heptanal	111-71-7	1179.2	8.60±1.80[a]	16.05±8.08[a]	9.50±1.49[a]	8.16±1.65[a]	13.31±5.58[a]
Octanal	124-13-0	1284	5.97±1.07[b]	33.72±13.96[a]	10.94±1.79[b]	9.97±1.67[b]	12.84±1.50[b]
2-Methyl-2-butenal	1115-11-3	1086.1	4.56±0.72[a]	4.93±1.95[a]	2.65±0.10[b]	n.d[c]	n.d[c]

5-Ethylcyclopent-1-enecarboxaldehyde	36431-60-4	1409.2	2.31±0.88[b]	n.d[c]	n.d[c]	3.64±0.13[a]	n.d[c]
(*E,E*)-2,4-Decadienal	25152-84-5	1807	1.05±0.97[b]	4.98±1.87[a]	n.d[b]	n.d[b]	n.d[b]
Decanal	112-31-2	1496.8	1.04±0.20[b]	6.95±1.93[a]	3.53±0.35[ab]	6.34±5.50[a]	3.91±0.75[ab]
(*E*)-2-Decenal	3913-81-3	1641.2	0.34±0.29[b]	2.05±0.41[a]	n.d[b]	n.d[b]	n.d[b]
Propanal	123-38-6	787.6	0.62±0.26[b]	2.12±0.92[a]	n.d[b]	2.47±0.21[a]	2.93±0.80[a]
(*E,Z*)-2,4-Decadienal	25152-83-4	1762.1	0.19±0.17[ab]	0.80±0.79[a]	n.d[b]	n.d[b]	n.d[b]
2-Undecenal	2463-77-6	1749.4	0.28±0.12[bc]	n.d[c]	1.09±0.18[a]	0.77±0.67[ab]	n.d[c]
1H-Pyrrole-2-carboxaldehyde	1003-29-8	2018.2	0.07±0.03[b]	0.39±0.08[ab]	0.57±0.06[ab]	0.47±0.12[ab]	1.85±1.85[a]
2-Thiophenecarboxaldehyde	98-03-3	1687.2	0.06±0.01[a]	n.d[b]	n.d[b]	n.d[b]	n.d[b]
Furfural	98-01-1	1457.2	n.d[c]	24.19±4.02[c]	65.16±26.76[b]	n.d[c]	286.39±33.28[a]
Methional	3268-49-3	1448.5	n.d[c]	20.94±2.31[b]	15.66±15.70[bc]	16.56±5.15[b]	49.79±9.84[a]
5-Methyl-2-furaldehyde	620-02-0	1567.7	n.d[c]	1.98±0.74[bc]	4.71±1.42[b]	n.d[c]	28.76±4.98[a]
Cumaldehyde	122-03-2	1775.2	n.d[b]	0.63±0.55[b]	3.49±1.03[a]	n.d[b]	n.d[b]
Aubepine	123-11-5	2017.4	n.d[b]	0.27±0.07[a]	n.d[b]	0.34±0.08[a]	0.25±0.07[a]
(*E*)-2-Nonenal	18829-56-6	1531.6	n.d[c]	n.d[c]	2.81±1.23[a]	n.d[c]	1.26±0.35[b]
Acetaldehyde	75-07-0	700.1	n.d[b]	n.d[b]	n.d[b]	37.90±8.16[a]	n.d[b]
2,4-Decadienal	2363-88-4	1805.9	n.d[b]	n.d[b]	n.d[b]	5.48±4.75[a]	3.52±0.66[ab]
α-Ethylidenbenzeneacetaldehyde	4411-89-6	1924.8	n.d[b]	n.d[b]	n.d[b]	0.86±0.30[a]	0.23±0.11[b]
2-Propenal	107-02-8	836.3	n.d[b]	n.d[b]	n.d[b]	0.32±0.37[a]	n.d[b]
Butanal	123-72-8	868.4	n.d[b]	n.d[b]	n.d[b]	0.29±0.15[a]	n.d[b]
(*E*)-2-Undecenal	53448-07-0	1748.9	n.d[b]	n.d[b]	n.d[b]	n.d[b]	0.63±0.56[a]
2-Formyl-1-methylpyrrole	1192-58-1	2096.1	n.d[b]	n.d[b]	n.d[b]	n.d[b]	0.32±0.06[a]
(*E*)-2-Pentenal	1576-87-0	1122.3	n.d[b]	n.d[b]	n.d[b]	n.d[b]	0.21±0.19[a]
5-Hydroxymethylfurfural	67-47-0	2494	n.d[b]	n.d[b]	n.d[b]	n.d[b]	0.07±0.06[a]
5-Methyl-2-thiophenecarboxaldehyde	13679-70-4	1803.4	n.d[b]	n.d[b]	n.d[b]	n.d[b]	0.07±0.02[a]

**Acids**							
Hexanoic acid	142-62-1	1848.5	4.37±0.56^{ab}	n.d^{b}	n.d^{b}	8.96±1.38^{a}	8.41±7.55^{a}
Isovaleric acid	503-74-2	1670.6	2.67±0.36^{b}	n.d^{c}	n.d^{c}	n.d^{c}	n.d^{b}
Propanoic acid	79-09-4	1538.6	1.34±1.16^{a}	n.d^{b}	n.d^{b}	n.d^{b}	n.d^{b}
Butanoic acid	107-92-6	1630.1	1.24±0.06^{ab}	n.d^{b}	n.d^{b}	n.d^{b}	1.62±1.43^{a}
Pentanoic acid	109-52-4	1741	0.75±0.02^{c}	1.44±0.27^{b}	n.d^{d}	n.d^{d}	2.27±0.38^{a}
Octanoic acid	124-07-2	2066.3	0.49±0.07^{a}	1.83±0.20^{a}	1.13±0.43^{a}	1.19±1.04^{a}	1.06±1.04^{a}
Heptanoic acid	111-14-8	1958.3	0.34±0.03^{cd}	1.62±0.16^{a}	n.d^{d}	0.73±0.65^{bc}	1.24±0.18^{ab}
Isobutylacetic acid	646-07-1	1806	0.33±0.06^{b}	n.d^{c}	n.d^{c}	n.d^{c}	0.99±0.10^{a}
Nonanoic acid	112-05-0	2174.8	0.14±0.03^{c}	0.98±0.24^{ab}	1.13±0.27^{a}	1.35±0.21^{a}	0.73±0.15^{b}
Acetic acid	64-19-7	1446.1	n.d^{b}	172.42±43.23^{a}	n.d^{b}	n.d^{b}	n.d^{b}
**Terpene**							
Linalool	78-70-6	1548.8	2.41±2.14^{c}	55.06±31.59^{b}	204.16±3.24^{a}	34.74±13.25^{b}	3.01±2.12^{c}
Eucalyptol	470-82-6	1200.3	38.90±6.02^{b}	2797.84±819.01^{a}	2326.23±215.15^{a}	12.28±3.53^{b}	4.26±0.38^{b}
Limonene	138-86-3	1190.9	6.37±5.71^{c}	2034.58±353.05^{a}	789.27±73.40^{b}	728.30±141.27^{b}	14.53±2.52^{c}
β-Myrcene	123-35-3	1157.6	0.84±0.94^{c}	394.82±78.90^{a}	210.16±26.10^{b}	175.61±45.88^{b}	3.73±0.81^{c}
α-Pinene	80-56-8	1015.8	1.96±0.30^{b}	n.d^{b}	570.04±75.35^{a}	39.03±12.33^{b}	2.56±0.57^{b}
Sabinene	3387-41-5	1105.8	1.21±1.35^{b}	483.83±154.53^{a}	n.d^{b}	19.36±6.68^{b}	0.53±0.12^{b}
γ-Terpinene	99-85-4	1235	0.22±0.19^{c}	299.39±60.47^{a}	61.36±5.66^{b}	8.09±1.17^{c}	n.d^{c}
Terpinen-4-ol	562-74-3	1601.8	0.14±0.12^{c}	81.25±26.15^{b}	165.61±5.36^{a}	n.d^{c}	n.d^{c}
3-Carene	13466-78-9	1134	0.12±0.14^{b}	n.d^{b}	1.96±0.66^{a}	n.d^{b}	1.50±0.56^{a}
α-Terpinene	99-86-5	1176.1	0.37±0.27^{b}	79.81±71.76^{a}	8.80±8.88^{b}	n.d^{b}	n.d^{b}
D-Limonene	5989-27-5	1194.8	n.d^{b}	1684.35±657.68^{a}	101.81±62.83^{b}	79.69±22.52^{b}	7.30±2.10^{b}
β-Thujene	28634-89-1	1022	n.d^{b}	252.80±73.75^{a}	n.d^{b}	n.d^{b}	n.d^{b}
Terpinolene	586-62-9	1275	n.d^{c}	115.88±20.28^{a}	28.67±2.98^{b}	4.85±1.07^{c}	0.43±0.20^{c}

cis-Geraniol	106-25-2	1800	n.d[b]	55.62±48.40[a]	0.36±0.32[b]	n.d[b]	n.d[b]
(1S)-2,6,6-Trimethylbicyclo[3.1.1]hept-2-ene	7785-26-4	1015.8	n.d[b]	62.62±24.00[a]	n.d[b]	n.d[b]	n.d[b]
α-Phellandrene	99-83-2	1153.5	n.d[c]	39.75±10.26[a]	13.70±3.45[b]	0.86±0.15[c]	0.36±0.20[c]
β-Pinene	127-91-3	1090.6	n.d[c]	25.20±6.02[b]	15.94±3.03[b]	54.00±17.29[a]	n.d[c]
cis-β-Ocimene	3338-55-4	1247.9	n.d[c]	14.49±3.75[b]	2.00±0.23[c]	33.38±7.22[a]	0.36±0.35[c]
p-Mentha-1,5,8-triene	21195-59-5	1240.5	n.d[b]	6.82±5.92[a]	n.d[b]	3.59±0.75[ab]	n.d[b]
trans-β-Ocimene	3779-61-1	1230.1	n.d[b]	6.27±3.27[b]	3.76±0.47[b]	235.10±57.09[a]	n.d[b]
β-Elemene	515-13-9	1587.3	n.d[b]	3.84±1.55[ab]	n.d[b]	4.96±4.32[a]	n.d[b]
trans-Carveol	1197-07-5	1835.6	n.d[b]	2.85±1.12[a]	3.63±1.19[a]	n.d[b]	n.d[b]
Camphore	464-48-2	1509.2	n.d[b]	1.33±0.42[b]	1290.29±138.26[a]	n.d[b]	n.d[b]
Nerolidol	7212-44-4	2041	n.d[b]	1.28±0.60[a]	n.d[b]	0.25±0.22[b]	n.d[b]
Calamenene	483-77-2	1829.1	n.d[c]	0.61±0.53[c]	19.40±2.85[b]	44.36±9.61[a]	n.d[c]
Caryophyllene	87-44-5	1593.8	n.d[c]	0.38±0.33[c]	192.67±12.40[a]	56.21±53.90[b]	n.d[c]
Hotrienol	29957-43-5	1772.8	n.d[b]	0.19±0.17[a]	n.d[b]	n.d[b]	n.d[b]
Camphol	507-70-0	1702.8	n.d[b]	n.d[b]	1485.42±169.52[a]	n.d[b]	n.d[b]
δ-Terpineol	7299-42-5	1673.4	n.d[b]	n.d[b]	330.21±18.26[a]	n.d[b]	n.d[b]
Camphene	79-92-5	1056.3	n.d[b]	n.d[b]	93.91±10.36[a]	n.d[b]	n.d[b]
Humulene	6753-98-6	1674.2	n.d[c]	n.d[c]	48.33±1.63[a]	16.31±3.43[b]	n.d[c]
β-Phellandrene	555-10-2	1197.8	n.d[b]	n.d[b]	26.17±22.76[a]	n.d[b]	n.d[b]
Levomenthol	2216-51-5	1641.3	n.d[b]	n.d[b]	22.01±19.64[b]	3168.99±1947.67[a]	n.d[b]
Isopulegol	89-79-2	1571	n.d[c]	n.d[c]	22.78±2.85[b]	45.16±11.34[a]	n.d[c]
Copaene	3856-25-5	1491.3	n.d[b]	n.d[b]	17.72±5.46[a]	n.d[b]	n.d[b]
Myrtenol	515-00-4	1792.4	n.d[b]	n.d[b]	13.15±1.36[a]	n.d[b]	n.d[b]
Fenchol	1632-73-1	1583.4	n.d[b]	n.d[b]	12.74±1.56[a]	n.d[b]	n.d[b]

γ-Cadinene	39029-41-9	1757.4	n.d[b]	n.d[b]	6.60±5.72[a]	n.d[b]	n.d[b]
β-Bisabolene	495-61-4	1725.3	n.d[b]	n.d[b]	9.83±1.42[a]	n.d[b]	n.d[b]
cis-Sabinol	3310-02-9	1805.9	n.d[c]	n.d[c]	8.55±0.67[a]	2.76±0.70[b]	n.d[c]
(*E*)-Pinocarveol	547-61-5	1654.5	n.d[b]	n.d[b]	5.77±0.30[a]	n.d[b]	n.d[b]
α-Calacorene	21391-99-1	1912.4	n.d[c]	n.d[c]	5.13±2.00[b]	8.20±1.85[a]	n.d[c]
α-Curcumene	644-30-4	1770.7	n.d[b]	n.d[b]	4.52±0.80[a]	n.d[b]	n.d[b]
Anethole	104-46-1	1821.7	n.d[b]	n.d[b]	2.24±1.94[a]	1.43±0.38[ab]	2.44±0.19[a]
p-Mentha-1,8-dien-7-ol	536-59-4	2004	n.d[b]	n.d[b]	0.69±0.14[a]	n.d[b]	n.d[b]
Neo-Menthol	491-01-0	1598.1	n.d[b]	n.d[b]	n.d[b]	396.00±179.01[a]	n.d[b]
δ-Cadinene	483-76-1	1756.6	n.d[b]	n.d[b]	n.d[b]	138.46±33.63[a]	n.d[b]
Germacrene D	23986-74-5	1709.3	n.d[b]	n.d[b]	n.d[b]	96.54±29.48[a]	n.d[b]
Lavandulol	498-16-8	1678.9	n.d[b]	n.d[b]	n.d[b]	84.36±30.13[a]	n.d[b]
(-)-β-Bourbonene	5208-59-3	1516.3	n.d[b]	n.d[b]	n.d[b]	63.35±4.28[a]	n.d[b]
γ-Muurolene	30021-74-0	1687.1	n.d[c]	n.d[c]	15.01±1.68[b]	46.22±11.76[a]	n.d[c]
α-Terpineol	98-55-5	1698.3	n.d[b]	n.d[b]	n.d[b]	35.89±7.49[a]	0.45±0.41[b]
(*E*)-β-Famesene	18794-84-8	1667.2	n.d[b]	n.d[b]	n.d[b]	10.85±9.58[a]	n.d[b]
α-Cubebene	17699-14-8	1536.7	n.d[b]	n.d[b]	n.d[b]	4.39±4.22[a]	n.d[b]
Bicyclogermacren	24703-35-3	1733.4	n.d[b]	n.d[b]	n.d[b]	6.22±2.03[a]	n.d[b]
β-Patchoulene	514-51-2	1696.3	n.d[b]	n.d[b]	n.d[b]	2.27±0.98[a]	n.d[b]
β-Selinene	17066-67-0	1716.4	n.d[b]	n.d[b]	n.d[b]	1.59±0.95[a]	n.d[b]
α-Corocalene	20129-39-9	2058.7	n.d[b]	n.d[b]	n.d[b]	0.99±0.24[a]	n.d[b]
1,3-Cyclopentadiene	542-92-7	733.8	n.d[b]	n.d[b]	n.d[b]	0.28±0.29[a]	n.d[b]
T-Muurolol	19912-62-0	2232.1	n.d[b]	n.d[b]	n.d[b]	0.08±0.02[a]	n.d[b]
Citronellol	106-22-9	1800	n.d[b]	n.d[b]	n.d[b]	n.d[b]	2.73±0.18[a]
α-Thujene	2867-05-2	1021.9	n.d[b]	n.d[b]	n.d[b]	n.d[b]	0.37±0.32[a]

Carvacrol	499-75-2	2210.4	n.d^{b}	1.23±0.54^{b}	20.73±3.75^{a}	n.d^{b}	n.d^{b}
Eugenol	97-53-0	2162.3	n.d^{b}	n.d^{b}	1.64±0.12^{a}	0.24±0.22^{b}	1.53±0.60^{a}
Neral	106-26-3	1677.9	0.50±0.26^{b}	4.67±1.15^{a}	n.d^{b}	n.d^{b}	n.d^{b}
α-Citral	141-27-5	1729	n.d^{b}	15.19±7.24^{a}	n.d^{b}	n.d^{b}	n.d^{b}
α-Campholenal	4501-58-0	1484.3	n.d^{b}	n.d^{b}	42.65±1.87^{a}	n.d^{b}	n.d^{b}
Myrtenal	564-94-3	1623.4	n.d^{b}	n.d^{b}	2.57±0.49^{a}	n.d^{b}	n.d^{b}
Piperitone	89-81-6	1726.2	n.d^{b}	n.d^{b}	n.d^{b}	824.85±173.94^{a}	n.d^{b}
p-Menthone	89-80-5	1487.4	n.d^{b}	n.d^{b}	n.d^{b}	778.43±340.35^{a}	n.d^{b}
Isopulegone	29606-79-9	1568.7	n.d^{b}	n.d^{b}	n.d^{b}	42.99±6.09^{a}	n.d^{b}
Thujone	546-80-5	1434.7	n.d^{b}	n.d^{b}	n.d^{b}	0.62±0.54^{a}	n.d^{b}
Caryophyllene oxide	1139-30-6	1980.9	n.d^{b}	n.d^{b}	1.43±0.17^{a}	n.d^{b}	n.d^{b}
Cadalene	483-78-3	2217.7	n.d^{b}	n.d^{b}	0.69±0.08^{a}	0.86±0.32^{a}	n.d^{b}
**Ketone**							
Acetone	67-64-1	810.9	47.61±7.59^{b}	105.24±24.08^{a}	n.d^{c}	76.01±28.24^{ab}	112.13±35.60^{a}
2-Heptanone	110-43-0	1177.7	10.72±2.44^{b}	18.30±1.45^{a}	19.20±2.57^{a}	19.29±4.06^{a}	16.52±3.19^{a}
Acetol	116-09-6	1292	8.32±1.14^{c}	20.10±4.53^{bc}	37.15±4.75^{a}	35.74±11.91^{a}	27.56±5.05^{ab}
Acetoin	513-86-0	1278.3	3.95±3.49^{bc}	n.d^{c}	8.77±1.94^{b}	8.66±2.65^{b}	15.80±5.98^{a}
2,3-Pentanedione	600-14-6	1056.5	3.90±0.66^{c}	7.63±1.61^{b}	n.d^{d}	7.35±1.82^{b}	10.98±1.68^{a}
5-Methyl-2-hexanone	110-12-3	1136.1	1.08±0.22^{b}	n.d^{c}	n.d^{c}	n.d^{c}	2.50±0.04^{a}
2-Hydroxy-3-pentanone	5704-20-1	1353.7	0.56±0.49^{bc}	0.50±0.43^{bc}	n.d^{c}	1.54±1.34^{ab}	2.33±0.46^{a}
6-Methyl-5-heptene-2-one	110-93-0	1333.5	0.81±0.06^{b}	13.13±5.63^{a}	13.00±0.59^{a}	n.d^{b}	4.82±2.10^{b}
Acetyl valeryl	96-04-8	1146.6	0.65±0.20^{a}	n.d^{b}	n.d^{b}	n.d^{b}	n.d^{b}
2(5H)-Furanone	497-23-4	1745.7	0.41±0.09^{c}	0.81±0.81^{bc}	n.d^{c}	2.11±0.56^{ab}	3.02±1.26^{a}
1-Hydroxy-2-butanone	5077-67-8	1366.9	0.19±0.17^{c}	n.d^{c}	0.77±0.15^{b}	1.38±0.54^{a}	1.72±0.27^{a}
2,3-Hexanedione	3848-24-6	1126.1	0.19±0.16^{ab}	n.d^{b}	n.d^{b}	n.d^{b}	0.57±0.50^{a}

3-Methyl-1,2-cyclopentanedione	765-70-8	1825	0.16±0.14[ab]	0.32±0.29[ab]	n.d[b]	n.d[b]	0.73±0.64[a]
2-Methyl-2-cyclopentenone	1120-73-6	1361.8	0.10±0.09[a]	n.d[b]	n.d[b]	n.d[b]	n.d[b]
Dihydro-2-methyl-3(2H)-furanone	3188-00-9	1258.4	n.d[b]	2.49±0.51[a]	n.d[b]	n.d[b]	n.d[b]
Melilotal	122-00-9	1769	n.d[b]	1.05±0.91[a]	n.d[b]	n.d[b]	n.d[b]
Furaneol	3658-77-3	2030.3	n.d[b]	1.53±0.24[ab]	2.39±2.07[ab]	4.07±2.05[a]	1.97±0.76[ab]
Isomenthone	491-07-6	1457.9	n.d[b]	n.d[b]	6.03±5.47[b]	2243.80±999.31[a]	n.d[b]
Dihydropseudoionone	689-67-8	1852.9	n.d[c]	n.d[c]	11.18±0.68[a]	3.35±3.02[b]	n.d[c]
3-Octanone	106-68-3	1250.4	n.d[c]	n.d[c]	9.20±1.60[a]	3.38±0.92[b]	n.d[c]
2-Octanone	111-13-7	1280.8	n.d[b]	n.d[b]	4.27±0.81[a]	n.d[b]	n.d[b]
Norfuronol	19322-27-1	2112	n.d[c]	n.d[c]	2.93±0.90[a]	n.d[c]	1.35±0.15[b]
Piperitone oxide	5286-38-4	1708	n.d[b]	n.d[b]	n.d[b]	84.26±6.18[a]	n.d[b]
2,3-Butanedione	431-03-8	969.7	n.d[b]	n.d[b]	n.d[b]	7.48±6.94[a]	n.d[b]
Pyranone	28564-83-2	2263.7	n.d[b]	n.d[b]	n.d[b]	3.64±1.20[a]	4.62±0.41[a]
cis-Jasmone	488-10-8	1940.9	n.d[b]	n.d[b]	n.d[b]	0.99±0.23[a]	n.d[b]
3,4-Hexanedione	4437-51-8	1135.7	n.d[b]	n.d[b]	n.d[b]	0.58±0.50[a]	n.d[b]
2-Hexanone	591-78-6	1004.2	n.d[b]	n.d[b]	n.d[b]	0.28±0.26[a]	n.d[b]
2-Pyrrolidinone	616-45-5	2034.3	n.d[b]	n.d[b]	n.d[b]	0.22±0.24[b]	0.48±0.13[a]
2,3-Octanedione	585-25-1	1322.2	n.d[b]	n.d[b]	n.d[b]	n.d[b]	7.29±0.78[a]
1-(6-Methyl-2-pyrazinyl)-1-ethanone	22047-26-3	1687.9	n.d[b]	n.d[b]	n.d[b]	n.d[b]	0.47±0.11[a]
Furyl hydroxymethyl ketone	17678-19-2	1998.1	n.d[b]	n.d[b]	n.d[b]	n.d[b]	0.23±0.21[a]
**Alkane**							
3-Methylnonane	5911-04-6	959.5	n.d[b]	3.36±2.94[a]	n.d[b]	0.38±0.33[b]	n.d[b]
Ethylcyclohexane	1678-91-7	882.2	1.69±0.47[a]	1.99±0.99[a]	n.d[b]	n.d[b]	n.d[b]
Undecane	1120-21-4	1089.2	0.84±0.23[a]	n.d[b]	n.d[b]	n.d[b]	n.d[b]
Dodecane	112-40-3	1195.5	0.53±0.53[b]	n.d[b]	n.d[b]	n.d[b]	2.23±1.94[a]

Tetradecane	629-59-4	1396.9	0.55±0.32[b]	n.d[b]	n.d[b]	2.85±2.68[a]	n.d[b]
Methyl Isobutyl Ketone	108-10-1	1004.2	0.27±0.25[ab]	n.d[b]	n.d[b]	n.d[b]	0.57±0.51[a]
2,2,4,6,6-Pentamethylheptane	13475-82-6	951.8	n.d[b]	20.47±7.91[a]	3.02±0.86[b]	2.73±1.59[b]	2.31±0.30[b]
Pentane	109-66-0	562.8	n.d[b]	n.d[b]	18.22±5.07[ab]	28.27±24.52[a]	22.18±1.55[a]
Octane	111-65-9	800.5	n.d[c]	n.d[c]	5.57±0.33[b]	8.29±3.63[b]	14.71±0.24[a]
Pentadecane	629-62-9	1500.6	n.d[b]	n.d[b]	0.67±0.02[a]	n.d[b]	n.d[b]
3-Nonanol	624-51-1	1495.7	n.d[b]	n.d[b]	n.d[b]	17.16±4.64[a]	n.d[b]
1-Methoxyoctane	929-56-6	1147	n.d[b]	n.d[b]	n.d[b]	5.18±0.96[a]	n.d[b]
Decane	124-18-5	1000.4	n.d[b]	n.d[b]	n.d[b]	1.32±0.50[a]	1.09±0.32[a]
3-Methyldecane	13151-34-3	1063.2	n.d[b]	n.d[b]	n.d[b]	n.d[b]	0.28±0.24[a]
**Esters**							
α-Terpinyl acetate	80-26-2	1700.2	11.11±13.36[b]	2413.89±2612.98[a]	n.d[b]	n.d[b]	9.54±0.89[b]
Actylol	97-64-3	1341.4	13.35±2.18[b]	20.91±1.49[b]	n.d[b]	8.19±7.29[b]	62.13±25.14[a]
Butyrolactone	96-48-0	1619.2	4.37±0.53[b]	8.51±0.99[ab]	14.64±2.22[a]	12.01±10.41[ab]	16.07±3.87[a]
Ethyl Acetate	141-78-6	883.5	3.30±0.41[b]	5.76±0.54[a]	2.30±2.26[b]	3.41±0.28[b]	6.12±1.21[a]
Octyl formate	112-32-3	1560.6	1.15±1.04[b]	18.42±16.06[a]	n.d[b]	n.d[b]	3.30±1.46[b]
Ethyl pyruvate	617-35-6	1265.6	0.79±0.71[ab]	n.d[b]	n.d[b]	1.80±1.57[a]	n.d[b]
Linalyl acetate	115-95-7	1555.6	1.14±0.63[b]	110.69±63.80[a]	n.d[b]	n.d[b]	n.d[b]
Methyl lactate	2155-30-8	1314.9	0.60±0.09[b]	0.94±0.21[b]	1.68±0.12[ab]	2.37±1.16[a]	2.64±0.96[a]
1-Methoxy-2-propyl acetate	108-65-6	1223.2	0.40±0.09[b]	0.51±0.05[a]	n.d[c]	n.d[c]	n.d[c]
Nerol acetate	141-12-8	1756.3	n.d[b]	32.10±13.43[a]	n.d[b]	n.d[b]	n.d[b]
Methyl 2-hydroxy-3-methylpentanoate	41654-19-7	1491.1	n.d[b]	26.05±5.32[a]	n.d[b]	n.d[b]	n.d[b]
Methyl formate	107-31-3	751.3	n.d[c]	8.20±2.50[b]	5.22±0.69[b]	6.97±2.43[b]	24.51±2.19[a]
Methyl 2-hydroxy-4-methylpentanoate	40348-72-9	1524.2	n.d[b]	6.29±1.72[a]	n.d[b]	n.d[b]	n.d[b]
Octyl acetate	112-14-1	1681.9	n.d[b]	6.13±2.29[a]	n.d[b]	6.87±1.17[a]	n.d[b]

Bornyl acetate	76-49-3	1579	n.d[b]	5.98±1.97[a]	n.d[b]	n.d[b]	n.d[b]
Methyl 2-hydroxy-3-methylbutanoate	17417-00-4	1397	n.d[b]	1.54±0.15[a]	n.d[b]	n.d[b]	n.d[b]
Ethyl caproate	123-66-0	1231.4	n.d[b]	0.87±0.75[a]	n.d[b]	n.d[b]	n.d[b]
(*E*)-Methyl cinnamate	1754-62-7	2072.6	n.d[b]	0.56±0.26[a]	n.d[b]	n.d[b]	n.d[b]
Dibutyl phthalate	84-74-2	2699	n.d[b]	0.28±0.05[a]	n.d[b]	n.d[b]	n.d[b]
2-Camphanol acetate	76-49-3	1579	n.d[b]	n.d[b]	103.10±1.77[a]	n.d[b]	n.d[b]
γ-Caprolactone	695-06-7	1694.6	n.d[b]	n.d[b]	2.89±0.06[a]	2.47±0.65[a]	n.d[b]
Menthyl acetate	89-48-5	1562.9	n.d[b]	n.d[b]	n.d[b]	1.31±0.44[a]	n.d[b]
Methyl acetate	79-20-9	821.7	n.d[c]	n.d[c]	n.d[c]	26.75±27.45[b]	86.49±9.54[a]
Hexyl isovalerate	10032-13-0	1446.5	n.d[b]	n.d[b]	n.d[b]	8.87±7.69[a]	n.d[b]
Isopulegyl acetate	89-49-6	1635.2	n.d[b]	n.d[b]	n.d[b]	6.27±2.42[a]	n.d[b]
Acetic acid ethenyl ester	108-05-4	891.2	n.d[c]	n.d[c]	n.d[c]	5.63±0.87[b]	9.32±2.88[a]
n-Valeric acid cis-3-hexenyl ester	35852-46-1	1472.9	n.d[b]	n.d[b]	n.d[b]	3.14±2.91[a]	n.d[b]
n-Decyl acetate	112-17-4	1681.9	n.d[b]	n.d[b]	n.d[b]	1.85±0.45[a]	n.d[b]
Methyl salicylate	119-36-8	1769.5	n.d[b]	n.d[b]	n.d[b]	1.31±0.44[a]	n.d[b]
γ-Heptanolactone	105-21-5	1796.1	n.d[b]	n.d[b]	n.d[b]	1.05±0.23[a]	n.d[b]
δ-Valerolactone	542-28-9	1795.5	n.d[b]	n.d[b]	n.d[b]	0.62±0.55[a]	n.d[b]
Ethyl α-methylbutyrate	7452-79-1	1050.2	n.d[b]	n.d[b]	n.d[b]	0.37±0.18[a]	n.d[b]
Methyl p-anisate	121-98-2	2086.8	n.d[b]	n.d[b]	n.d[b]	0.12±0.10[a]	n.d[b]
δ-Caprolactone	823-22-3	1784.1	n.d[b]	n.d[b]	n.d[b]	n.d[b]	0.11±0.09[a]
Tributyl phosphate	126-73-8	1917.7	n.d[b]	n.d[b]	n.d[b]	n.d[b]	5.09±0.77[a]
β-Phenethyl formate	104-62-1	1780.8	n.d[b]	n.d[b]	n.d[b]	n.d[b]	0.75±0.12[a]
Methyl butanoate	623-42-7	980.4	n.d[b]	n.d[b]	n.d[b]	n.d[b]	0.39±0.34[a]
Cyclohexyl isothiocyanate	1122-82-3	1665	n.d[b]	n.d[b]	n.d[b]	n.d[b]	0.39±0.04[a]
Dimethyl phthalate	131-11-3	2293.3	n.d[b]	n.d[b]	n.d[b]	n.d[b]	0.11±0.05[a]

**Others**							
Dimethyl disulfide	624-92-0	1064.5	4.03±1.33[a]	5.02±0.95[a]	1.87±9.72[b]	1.90±0.55[b]	1.51±0.34[b]
Dimethylformamide	68-12-2	1323.3	1.88±0.30[b]	4.10±1.06[a]	n.d[c]	4.56±0.49[a]	4.64±0.95[a]
Dimethyl sulfone	67-71-0	1891.5	0.78±0.68[d]	3.59±0.85[bc]	1.53±1.34[cd]	5.58±1.86[ab]	7.42±1.54[a]
Pyridine	110-86-1	1178.4	1.15±1.00[b]	n.d[b]	n.d[b]	3.75±1.50[a]	n.d[b]
Dimethyl trisulfide	3658-80-8	1372.3	1.03±0.62[b]	2.25±0.43[a]	n.d[c]	n.d[c]	n.d[c]
Isovaleronitrile	625-28-5	1119	0.91±0.07[ab]	n.d[c]	n.d[c]	0.65±0.57[b]	1.36±0.49[a]
Acetonitrile	75-05-8	998.7	0.58±0.53[b]	1.05±0.84[ab]	n.d[b]	2.41±0.71[a]	1.43±1.24[ab]
Thiazole	288-47-1	1244.6	0.78±0.15[a]	n.d[b]	n.d[b]	0.52±0.47[a]	0.72±0.05[a]
2-Acetylthiazole	24295-03-2	1642.4	0.69±0.13[b]	n.d[c]	n.d[c]	n.d[c]	1.85±0.24[a]
Tetramethylpyrazine	1124-11-4	1474.6	0.68±0.14[b]	1.23±1.09[ab]	1.42±0.25[ab]	2.40±0.97[a]	0.51±0.45[b]
Formamide	75-12-7	1779.3	0.44±0.03[b]	n.d[b]	n.d[b]	1.68±0.89[a]	1.79±0.12[a]
1-Methoxy-2-propanol	107-98-2	1129	0.27±0.24[ab]	0.46±0.41[a]	n.d[b]	n.d[b]	n.d[b]
2-Picoline	109-06-8	1214.7	0.40±0.11[b]	n.d[b]	n.d[b]	1.29±0.53[a]	n.d[b]
3-Thiotolene	616-44-4	1108.1	0.16±0.15[ab]	n.d[b]	n.d[b]	0.66±0.59[a]	n.d[b]
Indole	120-72-9	2437.2	0.19±0.08[b]	0.90±0.15[a]	0.15±0.07[b]	0.21±0.09[b]	0.06±0.01[b]
2-Acetylpyridine	1122-62-9	1597.5	0.14±0.04[a]	n.d[b]	n.d[b]	n.d[b]	n.d[b]
Estragole	140-67-0	1666.5	n.d[b]	9.94±4.39[a]	n.d[b]	n.d[b]	0.15±0.03[b]
2-Acetylpyrrole	1072-83-9	1966.4	n.d[c]	6.37±1.14[bc]	n.d[c]	8.63±7.63[ab]	14.40±4.10[a]
Ylangene	14912-44-8	1478.4	n.d[b]	0.75±1.01[b]	4.55±0.47[a]	n.d[b]	n.d[b]
2-Acetyl-2-thiazoline	29926-41-8	1751.5	n.d[b]	n.d[b]	2.27±0.49[a]	n.d[b]	2.06±0.13[a]
2-Octene	111-67-1	850.7	n.d[b]	n.d[b]	0.49±0.19[a]	n.d[b]	n.d[b]
2-Methylthiazole	3581-87-1	1233.9	n.d[b]	n.d[b]	n.d[b]	0.36±0.32[a]	n.d[b]
Trimethylamine	75-50-3	596	n.d[b]	n.d[b]	n.d[b]	5.43±5.60[a]	6.99±0.58[a]
Pyrrole	109-97-7	1510.3	n.d[c]	n.d[c]	n.d[c]	5.57±1.37[a]	4.04±0.66[b]

Dimethyl sulfide	75-18-3	741.7	n.d[b]	n.d[b]	n.d[b]	3.06±1.73[ab]	6.22±5.51[a]
Acetamide	60-35-5	1759.7	n.d[b]	n.d[b]	n.d[b]	1.76±0.69[a]	1.56±0.55[a]
Butyl diglycol	112-34-5	1794.1	n.d[b]	n.d[b]	n.d[b]	0.83±0.73[a]	n.d[b]
Carbon disulfide	75-15-0	724.3	n.d[c]	n.d[c]	n.d[c]	0.94±0.17[b]	1.90±0.68[a]
1-Methylpyrrole	96-54-8	1131.6	n.d[b]	n.d[b]	n.d[b]	0.16±0.16[a]	n.d[b]
Benzyl nitrile	140-29-4	1919.1	n.d[b]	n.d[b]	n.d[b]	n.d[b]	0.97±0.14[a]
Glycerin	56-81-5	2312.6	n.d[b]	n.d[b]	n.d[b]	n.d[b]	0.91±0.05[a]

Means with same letters within the same row are not significantly different at the 95% confidence level.

Different lower-case letters within the same row were the Duncan's multiple range tests carried out by using SPSS.

[e] Control represented smoked chicken wings without spices, CMW, RMW, MW and RW represented smoked chicken wings with cardamom, rosemary, mint and rose respectively.

Quantitative based on the peak area relative to the area of the IS.

n.d, not detected.

Table S2. Permutation test parameters of OPLS-DA models.

Comparison group	R^2X	R^2Y (p value)	Q^2 (p value)
Control vs CMW	0.835	1 ($p < 0.005$)	0.98 ($p < 0.005$)
Control vs RMW	0.878	1 ($p < 0.005$)	0.998 ($p < 0.005$)
Control vs MW	0.821	1 ($p < 0.005$)	0.994 ($p < 0.005$)
Control vs RW	0.791	1 ($p < 0.005$)	0.986 ($p < 0.005$)

Figure S1. OPLS-DA analysis results of volatile compounds in chicken wings before and after smoking with four spices. Figures A, C, E, and G are the two-dimensional scores plot of cardamom, rosemary, mint, and rose, respectively. Figures B, D, F, and H are the S-plot of cardamom, rosemary, mint, and rose, respectively.

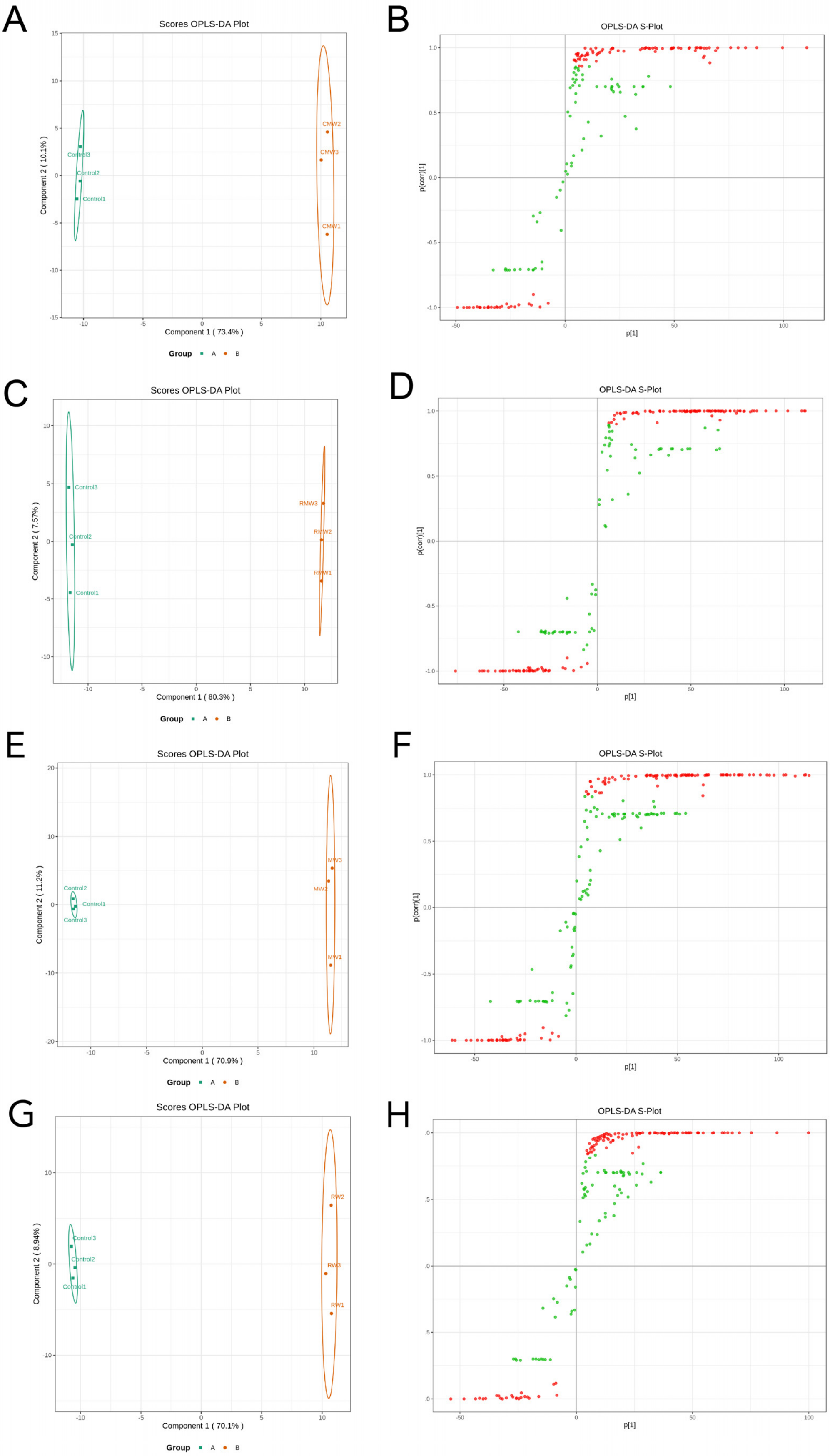
A
Scores OPLS-DA Plot
Component 1 (73.4%)
Component 2 (10.1%)
Control1
Control2
Control3
CMW1
CMW2
CMW3
Group A B
B
OPLS-DA S-Plot
p[1]
p(corr)[1]
C
Scores OPLS-DA Plot
Component 1 (80.3%)
Component 2 (7.57%)
Control1
Control2
Control3
RMW1
RMW2
RMW3
Group A B
D
OPLS-DA S-Plot
p[1]
p(corr)[1]
E
Scores OPLS-DA Plot
Component 1 (70.9%)
Component 2 (11.2%)
Control1
Control2
Control3
MW1
MW2
MW3
Group A B
F
OPLS-DA S-Plot
p[1]
p(corr)[1]
G
Scores OPLS-DA Plot
Component 1 (70.1%)
Component 2 (8.94%)
Control1
Control2
Control3
RW1
RW2
RW3
Group A B
H
OPLS-DA S-Plot
p[1]

Figure S2. Venn diagram of key differential volatile compounds in smoked chicken wings with different spices. The bar graph shows the number of key volatile compounds after smoked chicken wings with each spice.

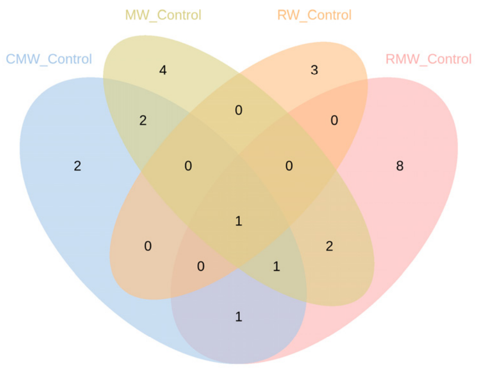

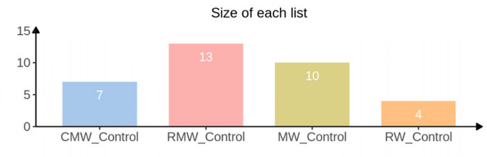

Supplement: Supplementary file 1 [file foods-14-02270-s001.zip › foods-3648675-supplementary.pdf]
